# Intravitreal Metformin Protects Against Choroidal Neovascularization and Light-Induced Retinal Degeneration

**DOI:** 10.3390/ijms252111357

**Published:** 2024-10-22

**Authors:** Jason F. Xiao, Wendy Luo, Amir Mani, Hugo Barba, Aniruddhsingh Solanki, Steven Droho, Jeremy A. Lavine, Dimitra Skondra

**Affiliations:** 1 Department of Ophthalmology and Visual Science, University of Chicago, Chicago, IL 60637, USA; jasonfxiao@uchicagomedicine.org (J.F.X.); luowendy@uchicago.edu (W.L.); amir.mani@bsd.uchicago.edu (A.M.);; 2 Animal Resources Center, University of Chicago, Chicago, IL 60637, USA; asolanki@bsd.uchicago.edu; 3 Department of Ophthalmology, Feinberg School of Medicine, Northwestern University, Chicago, IL 60611, USA; steven.droho@cuanschutz.edu (S.D.); jeremy.lavine@northwestern.edu (J.A.L.)

**Keywords:** age-related macular degeneration, choroidal neovascularization, retinal degeneration, metformin, intravitreal injection

## Abstract

Neovascular age-related macular degeneration (nAMD), a leading cause of blindness in older adults, presents a challenging pathophysiology involving choroidal neovascularization (CNV) and retinal degeneration. Current treatments relying on intravitreal (IVT) administration of anti-angiogenic agents are costly and of moderate effectiveness. Metformin, the common anti-diabetic drug, has been associated with decreased odds of developing AMD. Studies have shown that metformin can mitigate cellular aging, neoangiogenesis, and inflammation across multiple diseases. This preclinical study assessed metformin’s impact on vessel growth using choroidal explants before exploring IVT metformin’s effects on laser-induced CNV and light-induced retinal degeneration in C57BL/6J and BALB/cJ mice, respectively. Metformin reduced new vessel growth in choroidal explants in a dose-dependent relationship. Following laser induction, IVT metformin suppressed CNV and decreased peripheral infiltration of IBA1^+^ macrophages/microglia. Furthermore, IVT metformin protected against retinal thinning in response to light-induced degeneration. IVT metformin downregulated genes in the choroid and retinal pigment epithelium which are associated with angiogenesis and inflammation, two key processes that drive nAMD progression. These findings underscore metformin’s capacity as an anti-angiogenic and neuroprotective agent, demonstrating this drug’s potential as an accessible option to help manage nAMD.

## 1. Introduction

Age-related macular degeneration (AMD) is the most common cause of blindness in the United States for adults over 50 years of age and is a leading cause of blindness worldwide [1]. As the global population ages, the prevalence and disease burden of AMD is expected to rise; at least 8.7% of the global population is affected by AMD, with a projected prevalence of 288 million by 2040 [2]. Therefore, successful AMD treatment must not only be effective, but also accessible and economical in its delivery.

AMD can be categorized as “dry”, due to retinal degeneration and build-up of extracellular deposits, or neovascular (nAMD) due to growth of new blood vessels. Pathological angiogenesis is a significant feature of many retinal diseases [3], and nAMD in particular demonstrates choroidal neovascularization (CNV) [4]. AMD is classified into early, intermediate, and advanced stages based on clinical findings, including extent of subretinal deposits known as “drusen”, changes in the retinal pigment epithelium (RPE), and CNV [5]. Of those with AMD, an estimated 5–10% have nAMD, a subtype of advanced AMD, with the other patients having dry AMD [2,6,7]. Nevertheless, nAMD historically has accounted for 70–80% of cases of blindness caused by AMD [8]. The demarcation between dry AMD and nAMD, while useful clinically, is not mutually exclusive, as patients with nAMD also have underlying retinal atrophy [9]. Given AMD’s widespread prevalence, complex pathophysiology, and scarcity of effective treatment options, the disease remains at the forefront of ophthalmic research.

Recently, attention has turned to the so-called “wonder drug” metformin, a widely prescribed anti-hyperglycemic drug originally indicated for type 2 diabetes. Metformin has demonstrated remarkable potential to treat or prevent age-related diseases, such as cancer and cognitive decline [10,11,12]. In addition to its anti-inflammatory and antioxidant properties [13], metformin in certain contexts also inhibits angiogenesis [14]. Considering the drug’s pleiotropic effects, metformin has garnered great interest as a potentially effective and affordable treatment for both dry AMD and nAMD.

Multiple studies have retrospectively explored the relationship between oral metformin and AMD [15,16,17,18,19], primarily by assessing patient electronic health records or national insurance claims databases and recording oral metformin use as well as AMD occurrence. These studies showed that oral metformin may protect against AMD development in a dose-dependent relationship [19]. Although discovering these associations is a good first step, these studies are limited by their inability to prospectively examine metformin use and AMD development [20]. Furthermore, intravitreal (IVT) injection, a mainstay procedure for AMD patients, has not been thoroughly explored in the context of metformin delivery [21]. In animal models, IVT metformin has been shown to inhibit a senescence-associated secretory phenotype (SASP) and reduce retinal pathological vessel growth in oxygen-induced retinopathy, such as retinopathy of prematurity [22]. Furthermore, IVT metformin reduces the rate of apoptosis and markers of inflammation in *Rd1* mice, which model genetic retinal degenerative diseases such as retinitis pigmentosa [23]. While these findings are encouraging, how IVT metformin may impact choroidal neoangiogenesis remains unknown.

In this study, we investigated whether IVT metformin can protect against CNV and retinal degeneration, two key features of advanced AMD, and in the process explored changes in gene transcription and retinal morphology resulting from IVT administration.

## 2. Results

### 2.1. Metformin Suppresses Vessel Growth Area in a Dose-Dependent Manner in an Ex Vivo Model of Choroidal Sprouting

**Figure 1:**
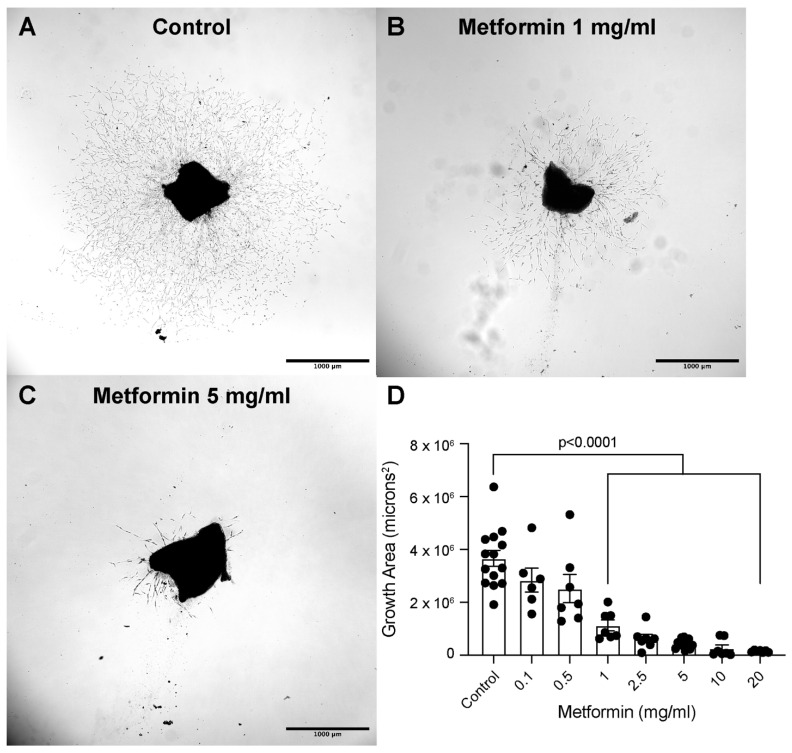
Metformin treatment significantly reduces choroidal sprouting area. Representative reverse phase brightfield images of control (**A**), 1 mg/mL (**B**), and 5 mg/mL metformin (**C**) treatment on Day 4; scale bar = 1 mm. Metformin reduced choroidal sprouting angiogenesis in a dose-dependent manner (**D**); *n* = 6–14 per group. Comparisons were made using Brown-Forsythe and Welch ANOVA followed by Dunnett’s T3 multiple comparisons test.

We first tested the effect of metformin on choroidal sprouting angiogenesis using the choroidal sprouting assay [24]. Metformin treatment resulted in a dose-dependent reduction in choroidal sprouting angiogenesis. Representative assay images of control, 1 mg/mL, and 5 mg/mL metformin are shown in Figure 1A–C. Metformin at concentrations of 0.1 mg/mL and 0.5 mg/mL did not significantly reduce choroidal growth area. However, higher concentrations of metformin reduced choroidal angiogenesis by 69.3%, 82.8%, 88.0%, 92.9%, and 96.1% at 1 mg/mL, 2.5 mg/mL, 5 mg/mL, 10 mg/mL, and 20 mg/mL, respectively, compared to controls (*p* < 0.001 for all versus control) (Figure 1D).

### 2.2. Intravitreal Metformin Does Not Significantly Change Retinal Morphology or Thickness

Once this dose-response relationship was established, we sought to study whether IVT metformin at the most concentrated dose tested induced any overt changes in retinal morphology or thickness that would suggest toxicity. Mice received either IVT 0.5 µL PBS or 0.5 µL of 20 μg/μL metformin, and after 7 days, the eyes were enucleated and stained with H & E. The retinas were vertically cross-sectioned using 10-μm slices around the optic nerve at distances of 100, 200, 300, and 500 μm in opposite directions.

**Figure 2:**
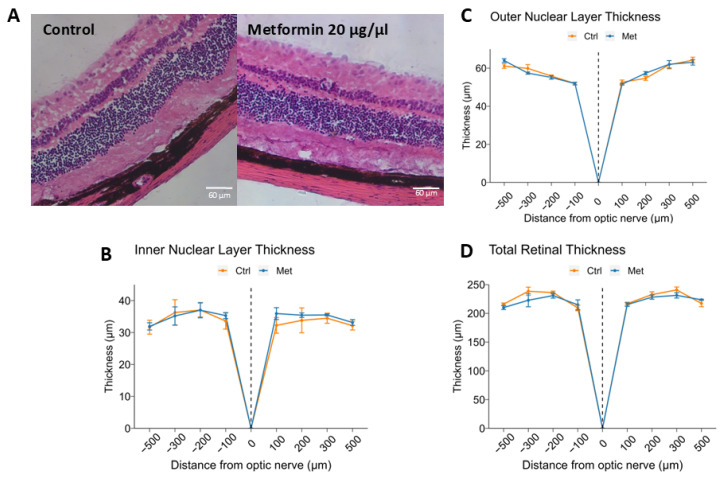
Effect of intravitreal metformin treatment on retinal morphology and thickness. 12-week-old, male C57Bl/6 mice received 0.5 μL intravitreal (IVT) administration of either vehicle control (PBS) or 20 μg/μL metformin (10 μg total) (*n* = 5 per group). After 7 days, eyes were enucleated, cross-sectioned, and stained with hematoxylin and eosin. Representative 20×-magnified images of vertical cross-sections are shown (**A**). The inner nuclear layer (INL) (**B**), outer nuclear layer (ONL) (**C**), and total retinal thickness (TRT) (**D**) were measured at four locations around the optic nerve: 100, 200, 300, and 500 µm in opposite directions. Eyes receiving IVT metformin had normal histology and similar retinal thicknesses without evidence of toxicity compared to eyes receiving vehicle control. Data are presented as mean ± SEM. Statistical testing was performed using Student’s t-test for comparison.

On microscopy, neither the metformin-treated nor control group presented with obvious morphological anomalies. H & E staining demonstrated that all retinal layers were present and well-defined (Figure 2A). Average thickness of the inner nuclear layer (INL) did not differ significantly between control and metformin-treated mice (31.1 ± 2.2 μm vs. 30.1 ± 2.2 μm, *p* = 0.773), nor did it differ individually at any of the locations analyzed (Figure 2B). Similarly, no differences were found between controls and metformin-treated mice with respect to outer nuclear layer (ONL) thickness (51.3 ± 2.8 μm vs. 51.4 ± 2.8 μm, *p* = 0.991), as well as total retinal thickness (TRT) (200.9 ± 14.1 μm vs. 197.5 ± 13.9 μm, *p* = 0.864) (Figure 2C,D).

### 2.3. Intravitreal Metformin Suppresses Choroidal Neovascularization and Infiltration of IBA1^+^ Macrophages/Microglia

**Figure 3:**
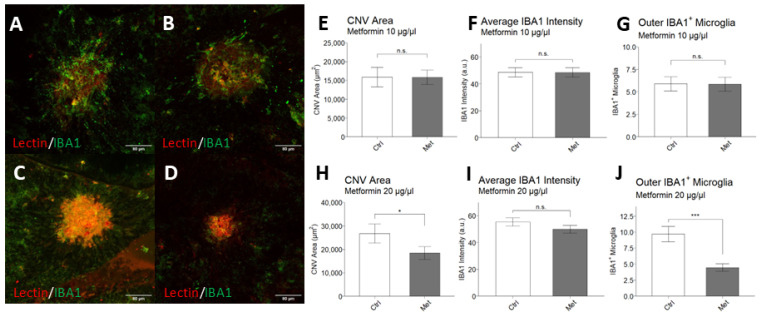
Intravitreal metformin suppresses laser-induced choroidal neovascularization (CNV) lesion size and IBA1^+^ microglia infiltration. Twelve-week-old male C57BL/6 mice underwent laser induction of CNV and immediate intravitreal (IVT) injection of vehicle control or metformin. Choroid/RPE flatmounts were made 7 days after the procedure and stained for Isolectin B4 (red) and IBA1 (green). Representative 20× confocal images of mice given IVT 0.5 μL sterile vehicle control (**A**,**C**), IVT 0.5 μL of 10 μg/μL metformin solution (5 μg total) (**B**), and IVT 0.5 μL of 20 μg/μL metformin solution (10 μg total) (**D**); scale bar = 80 μm. Lesion area (**E**), average IBA1 intensity (**F**), and number of IBA1^+^ macrophages/microglia (**G**) were compared between IVT metformin 5 μg and IVT vehicle. Lesion area (**H**), average IBA1 intensity (**I**), and number of IBA1^+^ macrophages/microglia (**J**) were compared between IVT metformin 10 μg and IVT vehicle. Experimental groups consisted of n = 16 mice per group; *n* = 4 lesions per eye. Data are presented as mean ± SEM. Statistical testing was performed using Student’s *t*-test for comparison; * *p* < 0.05, *** *p* < 0.001, n.s. = not significant.

After establishing a dose-response relationship of metformin on choroidal sprouting angiogenesis and confirming lack of histological evidence of toxicity, we sought to study whether IVT metformin affected CNV in vivo by using the laser-induced CNV mouse model of nAMD. Immediately after laser induction, mice received 0.5 µL of IVT vehicle control, 0.5 µL of IVT metformin at 10 µg/µL, or 0.5 µL of IVT metformin at 20 μg/µL (*n* = 16 mice per group). Representative images of choroid/RPE flatmounts are shown in Figure 3A–D. CNV lesion size was significantly decreased in mice that received 20 μg/µL IVT metformin compared to controls (18,434 ± 17,512 µm^2^ vs. 26,697 ± 28,470 µm^2^; *p* = 0.046) (Figure 3H). No significant differences in lesion size were detected between mice that received 10 μg/µL IVT metformin and their experimental controls (15,834 ± 13,144 µm^2^ vs. 15,903 ± 19,521 µm^2^; *p* = 0.492).

At the doses tested, IVT metformin did not significantly change signal intensity of IBA1 (Ionized calcium binding adaptor molecule 1) within the lesions (Figure 3F,I), a marker commonly used to generically identify macrophages/microglia of the innate immune system [25]. However, mice that received 0.5 µL of 20 µg/µL IVT metformin had fewer IBA1^+^ macrophages/microglia surrounding the CNV lesion compared to controls (4.48 vs. 9.70; *p* < 0.001) (Figure 3J). Between mice receiving 0.5 µL of 10 µg/µL IVT metformin and their controls, no differences were found in the quantity of IBA1^+^ macrophages/microglia surrounding the lesions (5.85 vs. 5.89; *p* = 0.485) (Figure 3G).

### 2.4. Intravitreal Metformin Alters RPE and Choroid Expression of Genes Related to Angiogenesis and Inflammation

**Figure 4:**
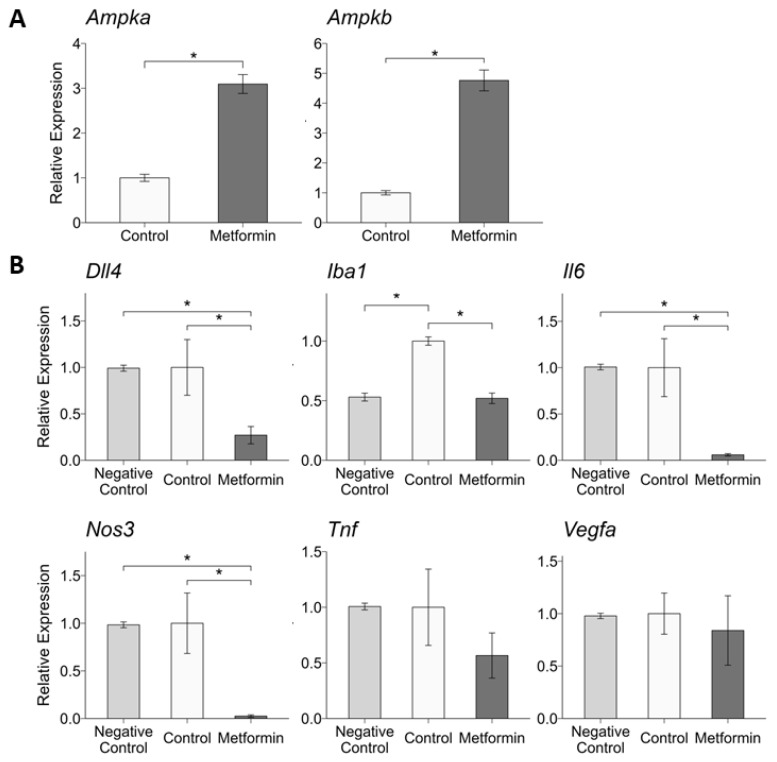
Intravitreal metformin treatment alters chorioretinal gene expression. Twelve-week-old male C57BL/6 mice received laser induction of choroidal neovascularization (CNV) and immediate intravitreal (IVT) injection of either 0.5 μL vehicle control or 20 μg/μL metformin solution equal to 10 μg total (*n* = 4 mice per group). Non-lasered mice (*n* = 6) were used as negative controls. Gene expression of *Ampka* and *Ampkb* (**A**), as well as *Dll4*, *Iba1*, *Il6*, *Nos3*, *Tnf*, and *Vegfa* (**B**) was assessed using qRT-PCR, with expression levels normalized to *Gapdh*. Data are presented as mean ± SEM. Statistical testing was performed using Student’s *t*-test for comparison; * *p* < 0.05.

To better understand potential mechanisms by which IVT metformin treatment resulted in decreased CNV size and fewer surrounding IBA1^+^ macrophages/microglia, RPE/choroid gene transcription was measured 7 days after laser induction and IVT metformin injection. In particular, genes implicated in nAMD pathogenesis and CNV were selected for investigation. The average RPE/choroid gene expression in mice treated with 0.5 µL of 20 µg/µL IVT metformin was normalized to controls (*n* = 4 mice per group). Non-lasered mice (*n* = 6) were used as negative controls. We first tested whether this dose of IVT metformin induced transcriptional changes of one of metformin’s most well-characterized downstream targets, *Ampk* (Adenosine Monophosphate-activated Protein Kinase). Specifically, we found that IVT metformin increased expression of both the alpha (*Ampka*) and beta (*Ampkb*) subunits of the enzyme (Figure 4A). Next, we compared the expression of ten target genes: *Dll4*, *Eglf7*, *Iba1*, *Il6*, *Nos3*, *Pgf*, *S100a8*, *Tie1*, *Tnf*, and *Vegfa* (Figure 4B and Supplemental Figure S1). Among these genes, IVT metformin decreased RPE/choroid expression of *Dll4* (Delta Like Canonical Notch Ligand 4), *Iba1*, *Il6* (Interleukin 6), and *Nos3* (Nitric Oxide Synthase 3). There were no statistically significant differences in expression of *Egfl7*, *Pgf*, *S100a8*, *Tie1*, *Tnf*, and *Vegfa* between the IVT metformin-treated mice and controls.

### 2.5. Intravitreal Metformin Protects Against Light-Induced Retinal Degeneration

**Figure 5:**
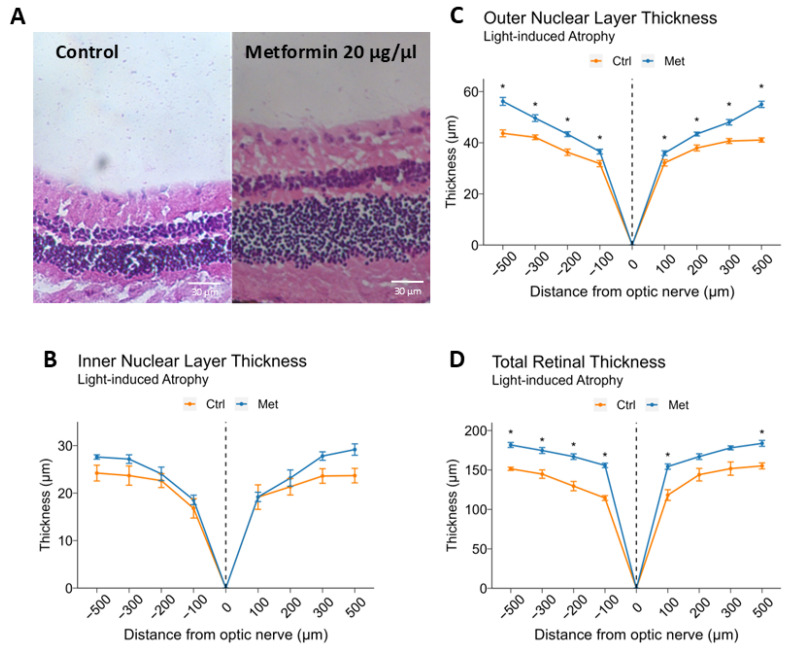
Intravitreal metformin treatment protects against light-induced retinal degeneration. Twelve-week-old, male BALB/cJ mice received 0.5 μL intravitreal (IVT) administration of either vehicle control (PBS) or metformin 20 μg/μL (total 10 μg) 6 h prior to dark adaptation. After 16 h of dark adaptation, mice were exposed to 2 h of light at an intensity of 7500 lux/hour (n = 5 per group). After 7 days, eyes were enucleated, cross-sectioned, and stained with hematoxylin and eosin. Representative 20×-magnified images of vertical cross-sections are shown (**A**). The inner nuclear layer (INL) (**B**), outer nuclear layer (ONL) (**C**), and total retinal thickness (TRT) (**D**) were measured at four locations around the optic nerve: 100, 200, 300, and 500 µm in opposite directions. Eyes receiving IVT metformin demonstrated thicker ONL and TRT compared to eyes receiving vehicle control. Data are presented as mean ± SEM. Statistical testing was performed using Student’s t-test for comparison; * *p* < 0.05.

Finally, we explored whether IVT metformin could protect against retinal degeneration induced by light exposure, a method commonly used for investigating atrophic, degenerative retinal diseases such as advanced dry AMD [26,27]. BALB/cJ mice received either IVT 0.5 µL PBS or 0.5 µL of 20 μg/μL metformin six hours prior to a 16-h period of dark adaptation. The mice were then exposed to 2 h of light at an intensity of 7500 lux/hour to induce retinal atrophy. Representative images of retinal cross-sections for both groups are shown in Figure 5A. Between IVT metformin-treated and control groups, average INL thickness did not significantly differ (21.9 ± 1.7 μm vs. 19.5 ± 1.5 μm, *p* = 0.294) (Figure 5B). On the other hand, the ONL was significantly thicker in the IVT metformin-treated group compared to controls (40.9 ± 1.7 μm vs. 34.0 ± 1.4 μm, *p* = 0.002) (Figure 5C). Consistent with this finding, TRT, while not statistically significant, was thicker in the IVT metformin-treated group (151.3 ± 10.7 μm vs. 123.2 ± 9.1 μm, *p* = 0.051).

## 3. Discussion

Over the past few decades, the potential to use metformin to prevent AMD or delay its progression has generated wide-spread interest in the scientific and medical community. Given the drug’s affordability, accessibility, and wide range of biological effects, metformin has been studied in various contexts, and its use has been thought to protect against several age-related processes, including cancer and cognitive decline [11,12,28]. In patients with type 2 diabetes, metformin has been shown to reduce ocular complications, such as diabetic retinopathy and glaucoma [2]. Specifically for AMD, retrospective studies have identified a dose-dependent effect between oral metformin and AMD development, though this relationship is not always identified depending on the dataset used [16,19,29]. Additionally, we have found in an nAMD mouse model that oral metformin decreases severity of CNV, affecting numerous transcriptional and metabolic pathways implicated in the disease’s pathogenesis [30]. However, it is not clear whether metformin’s impact results from the drug’s systemic and metabolic effects or through direct signaling in ocular tissue to regulate angiogenesis and inflammation. IVT drug therapy, a mainstay modality for treating nAMD, maximizes delivery of pharmacologic agents to ocular tissue while reducing systemic exposure [31]. Given its significance in real-world therapeutics for nAMD, exploring the possibility of IVT administration is warranted in preclinical pharmacologic research, as this method preserves in vivo biology and replicates established routes of administration. Furthermore, IVT administration of metformin directly assesses the drug’s effects in the retina and minimizes potentially confounding and pleiotropic effects of systemic metformin.

In this study, we determined that metformin decreases new vessel growth in choroidal explants in a dose-dependent relationship. Metformin has previously demonstrated anti-angiogenic properties in several disease states reliant on abnormal vessel growth, most notably cancer [32,33]. How metformin affects retinal vascular endothelium is under active investigation, with recent studies demonstrating metformin’s dose-dependent inhibition of angiogenesis in human retinal vascular endothelial cells in vitro and in vivo [34,35]. To our knowledge, the impact of metformin on choroidal vascular endothelial cells and angiogenesis remains unknown. Results from our ex vivo model substantiate the existing literature: metformin administration resulted in significantly reduced vessel growth area, with higher doses leading to higher reduction in growth area. This model serves as a successful proof-of-concept to demonstrate the suppressive effect of metformin on choroidal angiogenesis, the hallmark feature of nAMD.

After validating that metformin inhibited choroidal vessel growth, we assessed whether IVT metformin could reduce in vivo pathologic neovascularization in a mouse model of laser-induced CNV, the most widely used model of nAMD for therapeutic interventions and drug discovery [36]. Prior studies have examined the effects of intraperitoneal metformin injection on CNV [37] or the effects of IVT metformin on visual function in mice with retinal degeneration [23]. To our knowledge, the role of IVT metformin for nAMD lesions has not yet been explored in vivo. When mice received 0.5 μL of 10 μg/μL IVT metformin (5 μg total), CNV lesion size did not significantly change compared to controls. Oubaha and colleagues previously characterized a cellular state of senescence known as a senescence-associated secretory phenotype (SASP), which contributes to pathological retinal vessel growth and destructive angiogenesis in a model of retinopathy of prematurity/oxygen-induced retinopathy [22]. They found that a single 5 μg dose of IVT metformin reduced expression of several key SASP genes, such as *Il6*, *Cdkn1a*, and *Cdkn2a*, as well as decreased protein levels of IRE1α and NF-κB, highlighting the potential therapeutic effects of IVT metformin in retinopathy of prematurity. In our study, the inability of 0.5 μL of 10 μg/μL (5 μg total) IVT metformin to suppress CNV may indicate that lower doses of metformin may exert greater antiangiogenic effects in retinal vessels, rather than choroidal vessels, potentially through downregulating the SASP.

However, when we increased the dose of IVT metformin to 0.5 μL of 20 μg/μL (10 μg total), the resultant 31% decrease in average CNV lesion size became statistically significant. In addition to this anti-angiogenic effect, we found in our light-induced model of retinal degeneration that 0.5 μL of 20 μg/μL IVT metformin diminished retinal thinning compared to controls, particularly at the ONL. The ONL, composed of photoreceptor cell bodies and their nuclei, is an important anatomic endpoint for studies of retinal degeneration, advanced dry AMD, and geographic atrophy (GA) [38,39,40]. A combination of light-induced oxidative stress, inflammation, and disruption of the blood-retina barrier triggers photoreceptor apoptosis, resulting in thinning of the outer retina [41,42]. The finding that IVT metformin is neuroprotective in this light-induced model is consistent with the study by Luodan et al., in which 10 μg of IVT metformin rescued apoptotic photoreceptors, preserved ONL thickness, and delayed visual impairment in the *Rd1* mouse model of retinal degeneration [23]. Taken together, IVT metformin may offer both antiangiogenic and neuroprotective effects depending on the dose administered. Importantly, at this dose, we were unable to detect signs of toxicity on histology. These findings hold promise that IVT metformin may target both angiogenesis and retinal degeneration, two key features of nAMD, without compromising healthy tissue.

In addition to angiogenesis, dysregulated inflammation and innate immunity are other critical drivers of AMD, particularly through activation of resident tissue IBA1^+^ macrophages/microglia. Once injury occurs, microglia proliferate and migrate to lesion sites, creating a pro-inflammatory environment to facilitate repair and resolution of the insult [43,44]. However, with chronic conditions, such as AMD, this activation becomes pathologic, exacerbating retinal degeneration and encouraging neovascularization [45,46]. Besides regulating levels of endothelial growth factors, microglia alter the integrity of the blood-retina barrier, further impacting chorioretinal vasculature [47]. Subretinal microglia are also able to directly induce photoreceptor death and contribute to RPE atrophy [48]. Thus, treatments that can reduce inflammation from activated microglia are of great interest for retinal diseases, including nAMD. Our data show that only mice that received 0.5 μL of 20 μg/μL IVT metformin had significantly decreased numbers of subretinal IBA1^+^ macrophages/microglia around the lesions. Furthermore, IVT metformin attenuated the increase in *Iba1* transcription following laser induction. This finding matches the study by Luodan et al., in which IVT metformin suppressed IBA1^+^ macrophages/microglia throughout the retina, downregulating important inflammatory genes such as *Il10*, *Il4*, and *Tgfb1* in their *Rd1* mouse model of retinal degeneration [23]. Thus, IVT metformin may also decrease laser-induced CNV by dampening the inflammatory response and extent of macrophages/microglia infiltration.

To characterize potential biological mechanisms driving the protective effects of metformin, we measured RPE/choroid expression of various target genes following IVT metformin administration. Mice that received 0.5 μL of 20 μg/μL of IVT metformin showed a significant reduction in *Dll4* (Delta Like Canonical Notch Ligand 4) expression. In humans, DLL4 plays a crucial role in maintaining healthy retinal vasculature by coordinating the pro-angiogenic effects of VEGF during development [49]. However, overexpression of *Dll4* has been shown to be involved in pathological blood vessel growth in a murine model of oxygen-induced ischemic retinopathy [50]. Under hypoxic conditions, *Dll4* expression is increased following activation of Hif1α and Vegfa, and IVT administration of bevacizumab has been shown to reduce *Dll4* gene and protein expression in a rat model of CNV [51]. Furthermore, the study’s investigators found that Dll4 induced transcriptional changes in *Flt1*/*Vegfr1* and *Kdr*/*Vegfr2*, and that pharmacologic blockade of Notch signaling inhibited choroidal endothelial cell proliferation and migration. Studies in other disease states, including neonatal pulmonary hypertension and colorectal cancer, have identified a relationship with metformin and the downregulation of *Dll4* [52,53]. In our previous study looking at the role of oral metformin in reducing CNV, we did not find any statistically significant differences in *Dll4* expression in the RPE/choroid between control mice and mice given oral metformin [30]. However, oral metformin altered expression of *Notch1*, an important transcription factor in Notch signaling, suggesting this pathway could be involved in facilitating metformin’s protective effects. Furthermore, the finding that IVT metformin suppressed *Dll4* expression in the choroid/RPE could reflect a higher, targeted exposure of metformin through IVT administration, resulting in decreased CNV.

In addition to *Dll4*, we found that 10 μg IVT metformin reduced *Il6* expression. IL-6, a cytokine involved in instigating a pro-inflammatory response, is strongly implicated in AMD pathogenesis [54]. A recent meta-analysis indicates that systemic IL-6 levels are increased in patients with AMD, particularly in the advanced forms such as nAMD [55]. Furthermore, studies using human vitreous samples demonstrate increased IL-6 in the vitreous of patients with nAMD [22]. This is supported in animal studies, in which Droho et al. found that knocking out *Il6* significantly reduces CNV lesion area compared to wild-type mice [54]. In ex vivo choroidal sprouting assays, the investigators found that adding IL-6 to the media stimulated choroidal angiogenesis. Several groups have explored how metformin interacts with IL-6, and their results suggest that metformin may inhibit IL-6 signaling and reduce inflammation by decreasing expression of its receptor, IL-6R [56,57]. In agreement with our results, another study showed that IVT metformin can decrease *Il6* expression in mouse retinal tissue [22]. While the effect of IVT metformin on IL-6 signaling is not fully explored in our study, these findings are consistent with the literature in that IL-6 can promote nAMD, warranting further investigation.

We also found that IVT metformin significantly decreased expression of *Nos3* (Nitric Oxide Synthase 3). Nitric oxide is thought to stimulate endothelial VEGFA secretion and neoangiogenesis [58]. Additionally, as a reactive oxygen species, nitric oxide may directly damage endothelial cells and retinal tissue, leading to retinal degeneration and inflammation [59]. In a nAMD mouse model, CNV lesion size was found to more than double in mice receiving oral nitrite therapy compared to controls [60]. Previous studies have shown that metformin may increase nitric oxide levels in aortic and glomerular endothelial cells [61]. However, the effects of metformin on nitric oxide synthesis in the choroid and retina are not well-characterized, limiting the applicability of these findings to nAMD. Metformin in vitro can decrease expression of inducible nitric oxide synthase in primary human nonpigmented ciliary epithelial cells, resulting in decreased levels of nitric oxide in culture media [62]. We show that IVT metformin reduces *Nos3* expression in the RPE/choroid, which may partly explain the concomitant reduction in CNV lesion size. However, it remains unclear whether metformin’s modulation of nitric oxide synthesis and reactive oxygen species significantly affects CNV severity in our model.

We also examined how IVT metformin may affect expression of other pro-angiogenic genes linked to nAMD, such as *Vegfa*, *Tie1*, and *Eglf7*, and were unable to detect any significant differences in RPE/choroid expression for these target genes. Downregulation of *VEGFA* and other genes in the VEGF family following metformin administration has been demonstrated in several studies using cell lines, but this relationship remains inconclusive and under-explored in vivo [33,63]. Our results remain consistent with findings from other murine studies that were unable to demonstrate *Vegfa* reduction with metformin treatment [63,64]. With IVT metformin, despite decreasing pathologic angiogenesis, Oubaha et al. did not find increased expression of Vegf signaling at 5 μg metformin [22]. We also could not show a significant difference in levels of *Egfl7* (epidermal growth factor-like domain 7), an angiogenic signaling factor primarily expressed in endothelial cells that helps regulate *Vegfa* [65]. Additionally, *Tie1*, an endothelial cell tyrosine kinase essential for vessel maturation and downstream Vegf signaling [66], was not differentially expressed following IVT metformin. Considering that we administered a single dose of IVT metformin immediately after CNV laser induction, the lack of statistical significance in gene expression should be interpreted with caution. Some studies indicate that CNV formation peaks 5 days after laser induction and progressively declines [67], a timepoint at which we did not measure gene transcription. Furthermore, it is not clear when *Vegfa* mRNA levels peak, as studies indicate this may fall anywhere between 1–7 days post-laser [68,69,70]. Furthermore, one study showed that Vegfa protein levels are normalized 7 days after laser induction [69]. Our findings indicate that IVT metformin does not modulate angiogenesis through altering *Vegfa* transcription in this laser-induced CNV model, but rather downstream or through other regulatory mechanisms of angiogenesis. Nevertheless, further studies are needed to delineate this relationship.

Metformin is a potent activator of adenosine monophosphate-activated protein kinase (AMPK), which itself regulates a broad array of biological processes. In this study, we determined that one-time injection of IVT metformin upregulated transcription of *Ampka* and *Ampkb* in the RPE/choroid. As a highly conserved regulator of metabolism, AMPK is vital for cellular energy balance, homeostasis, and autophagy [71]. Through the AMPK pathway, metformin may decrease levels of inflammatory cytokines, including TNF, NRF2, MCP-1, IL-1B, and IL-6 [56,72,73,74]. Thus, AMPK activation may underlie the reduction in *Il6* expression that we observed, as well as other unmeasured inflammatory components that exacerbate CNV. In addition, metformin’s ability to reduce protein expression of DLL4 is thought to be mediated by AMPK [52]. Beyond the genes assessed in our study, metformin’s activation of AMPK can induce other changes that may regulate CNV, such as through inhibiting mTORc1, a protein complex that acts upstream of VEGF through HIF-1α [35,75]. We also found that IVT metformin attenuated light-induced retinal degeneration. Metformin has been shown to protect neuronal cells from various acute stressors, including ischemia, glucose deprivation, and accumulation of neurotoxic compounds through activating AMPK [76,77,78,79]. In addition, metformin’s activation of AMPK protects against oxidative stress and development of senescence, both critical to RPE cell functioning [80]. These protective effects may be mediated by AMPK’s regulation of transcription factors, such as FOXO, and deacetylases, such as SIRT1 and HDAC5, which generate broad downstream consequences [81]. Additionally, activation of AMPK by metformin has been shown to protect against drug-induced RPE cell death through reducing production of mitochondrial reactive oxygen species [82]. Thus, metformin’s ability to activate AMPK may contribute to its antiangiogenic and neuroprotective effects.

Anti-VEGF pharmacotherapy remains the first-line treatment for nAMD. Compared to IVT anti-VEGF injections, IVT metformin may offer advantages in several domains, including side-effect profile, cost, and alternative mechanisms of action. When drugs are administered intravitreally, they may enter systemic circulation through uveal vessel absorption and by aqueous humor outflow [83]. With anti-VEGF agents, systemic circulation may increase the risk of thromboembolic events, myocardial infarction, stroke, and hypertension among other adverse events due to disruptions in the regulation of proinflammatory genes [83,84]. Although systemic metformin can also cause adverse events, including metformin-associated lactic acidosis, these events are quite rare and are typically preceded by excessive plasma accumulation [85]. In addition, metformin influences multiple key biologic processes in retinal tissue, including inhibiting senescence, decreasing inflammation, and increasing expression of neuroprotective proteins such as crystallins [22,23]. Given that some patients develop resistance to anti-VEGF therapy, targeting distinct and separate pathways beyond VEGF may be critical in advancing nAMD management [86]. For instance, in patients with nAMD treated with IVT ranibizumab, an anti-VEGF agent, an estimated 66–76% will demonstrate recurrence after 12 months of treatment [87]. Metformin may also confer significant benefits in preventing progression of dry AMD or GA in patients with nAMD, as the drug has demonstrated promise in protecting against numerous neurodegenerative diseases through regulating processes including autophagy, mitochondrial biogenesis, and resistance to oxidative stress [88,89]. Multiple case-control studies have identified metformin use associated with decreased odds of developing dry AMD or GA [90,91]. Finally, anti-VEGF agents, which are biologics, are costly to manufacture and pose significant financial burden on patients [92,93]. Metformin, as a small molecule compound that is widely available, may be a more cost-effective and accessible solution [94]. Conversely, one of the major disadvantages of IVT metformin administration, especially compared to anti-VEGF, is that IVT metformin has not been well-studied. Thus, optimal dosing, frequency, and formulation are not known, as well as the magnitude of benefit compared to anti-VEGF or as an adjunct therapy. Similarly, in the laser-induced CNV mouse model, it is difficult to directly compare the effectiveness of IVT metformin with IVT anti-VEGF, as anti-VEGF agents such as bevacizumab do not consistently decrease CNV lesion size in mice [95,96]. This in part is thought to be due to bevacizumab’s poor cross-reactivity with murine VEGF, with approximately 5-log decrease in binding compared to human VEGF [97]. In one study with positive findings, a single injection of anti-VEGF resulted in approximately one-third decrease in CNV lesion size 14 days post-laser, which is comparable to our findings of IVT metformin decreasing CNV lesion size by 31% 7 days post-laser [98]. Nevertheless, future studies comparing IVT metformin to IVT anti-VEGF in models of nAMD are required to provide a more comprehensive understanding of IVT metformin as a novel treatment for AMD.

AMD is a multifactorial disease, with strong genetic and environmental risk factors contributing to its pathogenesis. The genetic contribution to AMD is strongly supported by the literature, with a 12- to 27-fold increase in developing the disease if a first-degree relative also has been diagnosed [99]. More than 50% of AMD’s heritability is linked to genetic variants at 34 loci associated with the complement system, extracellular matrix modeling, and lipid metabolism, such as *CFH* and *ARMS2*/*HTRA1* [100]. These genetic studies, along with preclinical experiments, have paved the way for the development of therapies against AMD that target dysregulated complement signaling, with other biological pathways being evaluated in clinical trials [101]. In addition, several environmental risk factors have been consistently identified, including smoking, sun exposure, and diet. Smoking is thought to increase the risk for developing any AMD by 2- to 7-fold through introducing oxidative stress, promoting inflammation, and impairing local vasculature [102,103]. Similarly, excess sunlight can hasten retinal degeneration and is thought to elevate risk for AMD [104]. Lastly, diets rich in antioxidants, vegetables, and omega-3 fatty acids are associated with decreased risk of AMD [105,106], whereas high-fat and high-glycemic diets are associated with increased risk [107,108]. Thus, while intravitreal pharmacologic agents remain a first-line treatment for advanced AMD, the most effective strategy in disease management will likely include a personalized and multi-faceted approach for both prevention and treatment.

This study has several key limitations. First, the animal models of nAMD and retinal atrophy, while commonly used in ophthalmic research, may not faithfully translate the effects of IVT metformin in human disease states. This is particularly true for the BALB/cJ strain, which are albino mice with exaggerated responses to light-induced degeneration. It is also important to consider that the retinal atrophy following acute light exposure likely differs clinically and mechanistically compared to that seen in dry AMD. Therefore, the role of IVT metformin must be evaluated in models that better recapitulate features of dry AMD [109,110]. Additionally, our transcriptional results lack complementary data, such as protein expression, and they assessed mRNA levels at only one timepoint, which may fail to capture important mechanistic insights into the protective effects of IVT metformin. Furthermore, we cannot comment on broader transcriptional changes in response to IVT metformin given our targeted assessment of select genes. Lastly, while retinal layer thickness is frequently used to evaluate toxicity in drug- and light-induced retinal degeneration models, we did not correlate these measurements with functional studies, such as electroretinography.

In summary, we show that metformin can be safely administered intravitreally to diminish choroidal neovascularization and presence of innate immune cells, specifically subretinal IBA1^+^ macrophages/microglia around the lesion. At the molecular level, metformin induces transcriptional changes in genes associated with angiogenesis and inflammation, two key processes underlying AMD. Furthermore, at this dose, IVT metformin also may offer some neuroprotective effects without evidence of toxicity on histology. The mechanisms underlying these results remain unknown, and further studies are required to assess the spatial and temporal changes that are orchestrated in response to IVT administration of metformin in vivo, including activation and migration of macrophages/microglia throughout the layers of the retina. In addition, the findings from this pilot study must be validated in separate animal models to demonstrate biologic plausibility and likelihood of translation to human physiology. Nevertheless, the suppression of pathologic angiogenesis and simultaneous neuroprotection provided by metformin is particularly relevant for nAMD given the frequent co-existence of CNV with retinal atrophy or GA. Findings from this study demonstrate the potential for repurposing this economical and widely-available drug for AMD and its vision-threatening consequences.

## 4. Materials & Methods

### 4.1. Animals

Male wild-type C57BL/6J and BALB/cJ conventional specific pathogen-free (SPF) mice were purchased from the Jackson Laboratories at 11 weeks of age. The SPF mice were housed at the Animal Resource Center’s barrier facility at the University of Chicago. All animals were fed a regular diet and offered reverse osmosis-sterilized water ad libitum. Mice were housed following a standard 12:12 light-dark cycle. Mice were euthanized via CO_2_ chamber and cervical dislocation. Experiments were performed in accordance with the Association for Research in Vision and Ophthalmology’s (ARVO) statement for the use of animals in Ophthalmic and Vision Research and were approved by the Institutional Animal Care and Use Committee of the University of Chicago (Protocol #727557).

### 4.2. Choroidal Sprouting Assay

Male wild-type C57BL/6J were bred in-house and maintained at the Northwestern University Center for Comparative Medicine in a pathogen-free barrier environment. Choroidal sprouting assays were performed as previously described [111]. Briefly, eyes were enucleated and dissected into posterior eye cups containing retinal pigment epithelium (RPE), choroid, and sclera. The peripheral RPE-choroid-sclera complex was separated from the central complex, cut into 0.5 mm × 0.5 mm pieces, and plated into growth factor reduced Matrigel (#356231; Corning, Bedford, MA, USA) in a 48-well plate on ice. The Matrigel was solidified and incubated for 2 days in EGM2-MV medium (CC3202; Lonza, Walkersville, MD, USA). On Day 2, metformin at concentrations of 0, 0.1, 0.5, 1, 2.5, 5, 10, and 20 mg/mL was added. On Day 4, reverse phase brightfield microscopy was performed on a Nikon Ti2 Widefield microscope (Nikon Metrology, Brighton, MI, USA) using a 4× objective. Images were analyzed with the Nikon Elements General Analysis as previously described [111]. Comparisons between choroidal sprouting areas were made using Brown-Forsythe and Welch ANOVA followed by Dunnett’s T3 multiple comparisons test.

### 4.3. Laser-Induced Choroidal Neovascularization

At 12 weeks of age, C57BL/6J mice underwent laser-induced CNV, the most commonly used model for CNV and nAMD. Before the procedure, mice were anesthetized with intraperitoneal ketamine 80–100 mg/kg and xylazine 2.5–10 mg/kg. After confirming the absence of stimuli response through toe pinch, they underwent laser photocoagulation (4 spots at the 3, 6, 9, and 12 o’clock meridians around the optic nerve, power 120 mW/duration 0.1 s/50 µm spot size, Argon 532 nm, Iridex OcuLight GL). Formation of a bubble during laser photocoagulation was used to confirm rupture of Bruch’s membrane. Eyes with hemorrhage were excluded.

### 4.4. Intravitreal Injection

Immediately after the laser procedure, the mice were transferred to the microscope area. The pedal withdrawal reflex was evaluated to confirm that they were still under anesthesia. DPBS (14190144, Thermo Fisher Scientific, Waltham, MA, USA) vehicle control and metformin solutions were prepared in advance and sterilized using a 20 µm filter (SLGSR33SS, MilliporeSigma, Beijing, China). The anesthetized mice were placed horizontally on the dissecting microscope with their left eye facing up. Either 0.5 µL of 10 µg/µL or 20 µg/µL metformin (D150959, Sigma Aldrich, St. Louis, MO, USA) solution was then injected to achieve a total of 5 µg or 10 µg per eye, respectively. The right eye was injected with the vehicle control. For the intravitreal injection, a hole of about 1 mm posterior to the superior limbus was made using a sterile 30-gauge needle and immediately removed. A 33-gauge needle (7803-05, Hamilton, Reno, NV, USA) mounted onto a 2.5 μL glass syringe (7632-01, Hamilton, Reno, NV, USA) was utilized to inject 0.5 μL of solution (vehicle or metformin) through the created hole. The needle bevel, facing the administrator, was inserted at a 45-degree angle and advanced about 0.75 mm. Each mouse was injected in both eyes. The eyes were then coated with erythromycin USP 0.5% ointment (AB09232, Bausch & Lomb, Tampa, FL, USA). The mice were placed on a warm 32 °C plate for recovery. After the procedure, the mice were housed under standard conditions.

### 4.5. Choroid/RPE Flatmounts and Immunohistochemistry

After 7 days following the laser and intravitreal procedure, the mice were euthanized, and their eyes were enucleated and fixed with 4% paraformaldehyde overnight at 4 °C. Following fixation, the choroidal cup was separated and cut to form 4 petals. The cut cups were washed for 1 min in 1x TBS before being left overnight at 4 °C in a donkey serum blocking solution (5% donkey serum, 2.5% BSA, 0.5% Triton X-100 in 1× TBS). The next day, the cups were placed in a new tube containing fresh donkey serum blocking solution with 1:500 anti-rabbit IBA1 (FUJIFILM Wako Chemicals, Richmond, VA, USA, 6A4) and incubated overnight at 4 °C. The samples were then washed with six 10-minute cycles of 5% TBS-T (TBS 1×, 5% Tween-20). Next, the cups were incubated for 20 min at room temperature with FITC (1:400) before being placed in the dark with 1:100 *Griffonia Simplicifolia* Lectin I (B-1105-2, Vector Laboratories, Newark, CA, USA) for 2 h with gentle rocking. The cups underwent 6 more washes for 10 min each with 5% TBS-T (TBS 1×, 5% Tween-20) before being mounted on a glass slide using ProLong Gold antifade reagent (ThermoFisher Scientific, Waltham, MA, USA).

### 4.6. Visualization and Quantification of Choroid/RPE Flatmounts

Flatmounts were visualized using a Leica SP5 confocal microscope (Leica Microsystems, Deerfield, IL, USA) at 20× magnification. Max intensity Z-stack generated images were used to quantify lesion area and IBA1^+^ microphages/microglia signal intensity. The outline of the lectin channel was used to quantify the lesion areas. This outline was overlapped in the FITC-IBA1^+^ channel to calculate the average signal from the IBA1^+^ microphages/microglia. An area of 450 µm from the center of the lesion was determined, and IBA1^+^ microphages/microglia outside of the lesion and within the 450 µm boundary were manually counted. IBA1^+^ signal intensity was calibrated to background signal intensity of non-lasered RPE/choroid tissue. Images were processed in FIJI. Data were quantified by two independent and masked graders to ensure measurement reliability. Statistical testing was performed using Student’s *t*-test for comparison, with *p*-values < 0.05 considered significant.

### 4.7. RNA Extraction and Quantitative Real-Time Reverse Transcription-Polymerase Chain Reaction (qRT-PCR)

Mice were sacrificed 7 days following the laser and intravitreal procedure, and their eyes were immediately enucleated and dissected. Non-lasered mice were used as negative controls. RPE/choroid tissue was extracted under appropriate sterility and decontamination techniques with RNase ZAP (Thermo Fisher Scientific, Waltham, MA, USA) used on all surfaces and equipment. RNAlater (Qiagen, Hilden, Germany) was used to store tissue at −80 °C until RNA extraction. Four samples of purified RNA were collected for each group using the Rneasy Kit (Qiagen, Hilden, Germany). RNA concentration was confirmed for each sample using a NanoDrop 2000cc (Thermo Fisher Scientific, Waltham, MA, USA) before being sequenced.

To create the first strand of c-DNA, the APE×BIO HyperScript™ Reverse Transcriptase kit (Apexbio Technology, Houston, TX, USA) was utilized. Cycling was carried out in a Bio-Rad T100 Thermal cycler, with parameters set to 25 °C for 2 min, 45 °C for 50 min, and 75 °C for 15 min. Custom primers were used for each target gene (Supplementary Materials), and the qRT-PCR was completed using PrimeTime Gene Expression MasterMix (IDT) and SybrGreen. Samples were run in triplicate using the BIO-RAD CFX384 Real-Time System. Cycling parameters were set to 95 °C for 3 min; 40 cycles of 95 °C for 15 s and 60 °C for 60 s; and lastly, 4 °C for 7 min. *Gapdh* was used to normalize gene expression. Results were analyzed utilizing the Bio-Rad CFX Maestro software (version 1.4.2433.1219), and Student’s two-sample *t*-test was performed between the normalized expression levels of the control and metformin groups.

### 4.8. Light-Induction of Retinal Degeneration

At 12 weeks of age, male wild-type BALB/cJ mice were injected with either 0.5 µL of IVT vehicle control or 20 µg/µL metformin solution before being dark-adapted for 16 h. Mice were then pharmacologically dilated with 2.5% phenylephrine ophthalmic solution. Each mouse was then separately exposed to 2 h of light at 7500 lux in a standardized fashion. After 7 days, the mice were sacrificed, and their eyes were enucleated, stained, and cross-sectioned for histological analysis.

### 4.9. Histology

After 7 days since IVT injection, the eyes were enucleated, fixed with 4% paraformaldehyde, and placed in O.C.T. compound (Sakura Finetek, Torrance, CA, USA). Vertical cross sections 10 μm thick were taken around the optic nerve and stained with hematoxylin and eosin (H & E). The stained cross-sections were visualized under 20× magnification using an inverted microscope (Zeiss Axioskop Fluorescence Phase Contrast Microscope) and then analyzed in ImageJ (version 1.54k). Thicknesses of retinal layers were measured at 9 different locations: 0, 100, 200, 300 and 500 µm to the right of the optic nerve and 100, 200, 300 and 500 µm to left of the optic nerve. The total retinal thickness (TRT), outer nuclear layer (ONL), and inner nuclear layer (INL) were measured in ImageJ (version 1.54k), and Student’s two-sample *t*-test was performed to assess for differences in retinal layer thickness, with *p*-values < 0.05 considered significant.

## Data Availability

The data presented in this study are available on request from the corresponding author.

## Supplementary Materials

The following supporting information can be downloaded at: https://www.mdpi.com/article/10.3390/ijms252111357/s1.
