# Supplementary material for: Intravitreal Metformin Protects Against Choroidal Neovascularization and Light-Induced Retinal Degeneration
Source: Int J Mol Sci. 2024 Oct 22;25(21):11357. doi: 10.3390/ijms252111357 (PMC11545389; doi:10.3390/ijms252111357)
Supplement: Supplementary file 1 [file ijms-25-11357-s001.zip › Supplemental File 1.pdf]

- Dll4
    - F: AAGCAGGTTTCAGTAGCGGC
    - R: CGGTTATCTCCCGGTGCATT
- Eglf7
    - F: ACAGACCCAGCCGTAGAGTG
    - R: TCAATTCGGTCCAGCTGCTGG
- Il6
    - F: TCCTTCCAGCCGGTGAGATA
    - R: GAGTTGGTGGGTAAGGGAGC
- Nos3
    - F: GGCCTGAACTGAGCATGGAT
    - R: GATTAGGCTCTTCCTGCTCCC
- Pgf
    - F: GATCGCTAGGGTTCGTCCAG
    - R: GATGGGGAACAGACAAGGGG
- S100A8
    - F: GAGAAAGCGTGGGGAAGTCT
    - R: TTTGACCTGGCCTGTACCAC
- Tie1
    - AAGCAGACAGACGTGATCTGG
    - GCACGATGAGCCGAAAGAAG
- Tnf
    - F: AACTGTAAGCGGGGCAATCA
    - R: TGAGGGTCTGGGCCATAGAA
- Vegfa
    - F: CGTGTTGCTTGAGTGGGTAGG
    - R: CAGGGTTGTCACACTTGCCT
