# Supplementary figures and images for: Intravitreal Metformin Protects Against Choroidal Neovascularization and Light-Induced Retinal Degeneration
Source: Int J Mol Sci. 2024 Oct 22;25(21):11357. doi: 10.3390/ijms252111357 (PMC11545389; doi:10.3390/ijms252111357)

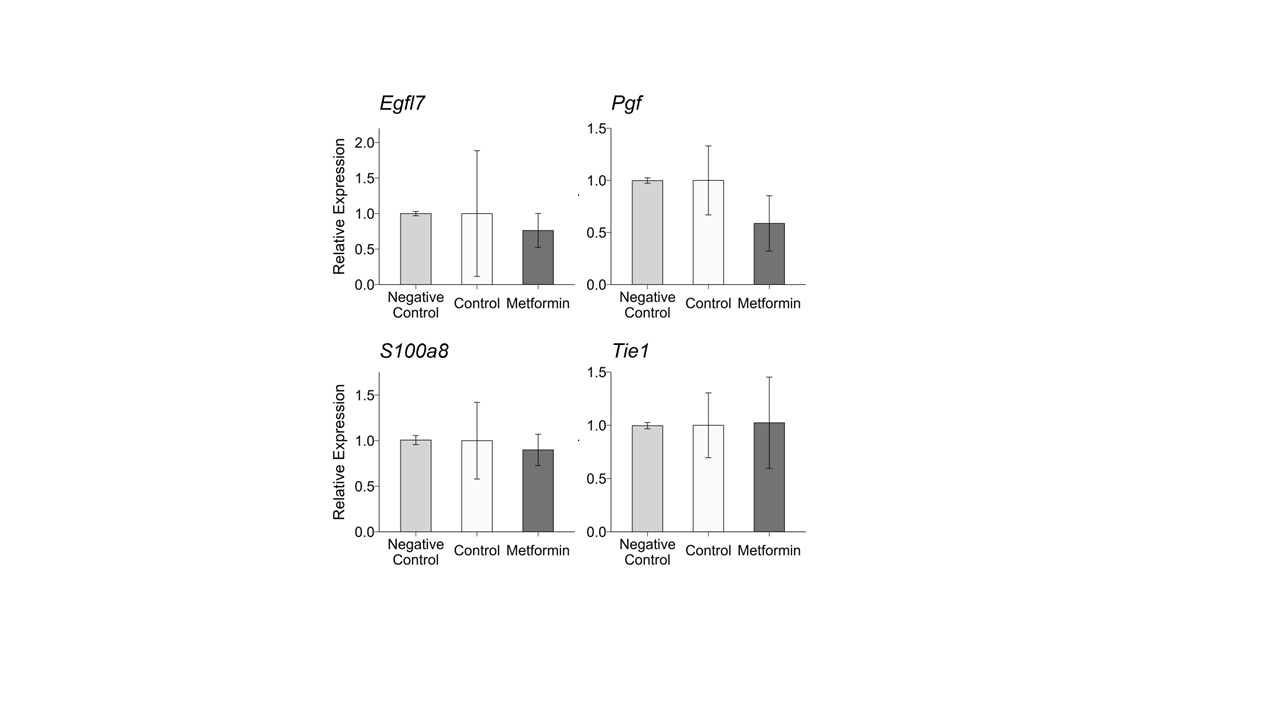

Supplement: Supplementary file 1 [file ijms-25-11357-s001.zip › Supplemental Figure S1.TIF]
